# The role of superior hemorrhoidal vein ectasia in the preoperative staging of rectal cancer

**DOI:** 10.3389/fonc.2024.1356022

**Published:** 2024-08-05

**Authors:** Nicola Maria Lucarelli, Alessandra Mirabile, Nicola Maggialetti, Chiara Morelli, Roberto Calbi, Simona Bartoli, Pasquale Avella, Domenico Saccente, Sara Greco, Antonio Amato Ianora Stabile

**Affiliations:** ^1^ Interdisciplinary Department of Medicine, Section of Diagnostic Imaging, University of Bari Medical School “Aldo Moro”, Bari, Italy; ^2^ Radiodiagnostic Complex Operating Unit, San Giacomo Hospital, Bari, Italy; ^3^ Radiology Unit, Ente Ecclesiastico Ospedale Generale Regionale “F. Miulli”, Bari, Italy; ^4^ Department of Clinical Medicine and Surgery, University of Naples “Federico II”, Naples, Italy

**Keywords:** computed tomography, CT, rectal cancer, superior hemorrhoidal vein, tumor diagnosis, prediction

## Abstract

**Objective:**

The prognosis of colorectal cancer has continuously improved in recent years thanks to continuous progress in both the therapeutic and diagnostic fields. The specific objective of this study is to contribute to the diagnostic field through the evaluation of the correlation between superior hemorrhoidal vein (SHV) ectasia detected on computed tomography (CT) and Tumor (T), Node (N), and distant metastasis (M) examination and mesorectal fascia (MRF) invasion in the preoperative staging of rectal cancer.

**Methods:**

Between January 2018 and April 2022, 46 patients with histopathological diagnosis of rectal cancer were retrospectively enrolled, and the diameter of the SHV was evaluated by CT examination. The cutoff value for SHV diameter used is 3.7 mm. The diameter was measured at the level of S2 during portal venous phase after 4× image zoom to reduce the interobserver variability. The parameters evaluated were tumor location, detection of MRF infiltration (defined as the distance < 1 mm between the tumor margins and the fascia), SHV diameter, detection of mesorectal perilesional lymph nodes, and detection of metastasis.

**Results:**

A total of 67.39% (31/46) of patients had SHV ectasia. All patients with MRF infiltration (4/46, 7.14%) presented SHV ectasia (average diameter of 4.4 mm), and SHV was significantly related with the development of liver metastases at the moment of primary staging and during follow-up.

**Conclusion:**

SHV ectasia may be related to metastasis and MRF involvement; therefore, it could become a tool for preoperative staging of rectal cancer.

## Introduction

Colorectal cancer (CRC) is the third most common cancer worldwide and the fourth most common cause of cancer-related death and, in Western countries, represents about 30% of large bowel cancers (1–5).

The CRC includes a dissimilar group of diseases in terms of mutations and mutagens, representing a challenging field for molecular therapy. Furthermore, the heterogeneity of embryological origins, anatomy, and functions underlines the differences between colon and rectal cancer. More than 30% of patients experience metastasis after primary tumor diagnosis, whereas peritoneal dissemination has long been associated with unfavorable prognosis (6–8).

Rectal cancer has a wide distribution from the seventh decade onward, although diagnoses in patients under 50 are increasing (9).

The median age at diagnosis is 70 years old, with an increase among frailty patients (10). However, many studies demonstrated rapidly increasing incidence rates among adults younger than 50 years (11, 12).

Accurate preoperative staging is mandatory to choose the most precise treatment strategy, taking into account the continuously rising rates of minimally invasive surgery (13–21). It is usually conducted through American Joint Committee on Cancer (AJCC) TNM classification (22, 23). Among the radiological features, the tumor infiltration pattern is strictly related to the patient’s prognosis (24). In particular, the invasion through the rectal wall, expressed by the T stage, is defined by imaging features in the pre-operative evaluation. T stage is related with local recurrence and has a role in the choice between up-front surgery and neo-adjuvant therapy (25–27).

In preoperative staging, rectal ultrasound endoscopy (EUS) is essential for early-stage tumors (T1 and T2). MRI is generally unable to distinguish T1 tumors (growing into the submucosa) from T2 tumors (growing into the muscularis propria) and is considered the standard tool for rectal cancer in more advanced stages (T3–T4) where accuracy in evaluating the infiltration of the mesorectal fascia (MRF) is fundamental.

As highly reported in the literature since the 1990s (28–30), in patients affected by locally advanced T3–T4 and/or N1–N3 low or middle rectal cancers or for tumors with circumferential margin < 1 mm regardless of the site and stage at magnetic resonance imaging (MRI) (31), preoperative radiotherapy followed by surgery represents the curative treatment.

According to the European Society of Medical Oncology guidelines, neoadjuvant chemotherapy is deserved to patients with a grade of infiltration > 5 mm at MRI evaluation (32, 33). MRI has a great sensitivity in the evaluation of T and N stages, approximately of 90%, and is the most accurate tool for the loco-regional staging of rectal cancer (34, 35).

Although radiological and surgical efforts to reduce the side effects of radiotherapy as proctitis, anal incontinence, anastomotic leak or stenosis, the optimal dose of radiotherapy is still debated (36–41).

In this clinical scenario, the most challenging stage to characterize with the standard imaging protocols is the T3, which is related to an overall 5-year survival ranging from 25% to 90%, depending on the T3 subgroup (32, 42–44).

A prognostic role has also been attributed to extramural vascular invasion (EMVI) and involvement of MRF representing poor prognostic factors (45–47). The EMVI is defined as the presence of tumor cells beyond the muscularis propria in endothelium-lined vessels (48, 49), and it is defined by the histological report as lymph-vascular invasion (LVI) (50). EMVI is reported as a risk factor for recurrent disease and metastasis and as a stage independent negative prognostic factor, increasing the risk of developing liver metastases (51, 52).

MRF is considered involved when the distance between the tumor and MRF is ≤1 mm. MRI has the highest accuracy concerning T and N stages and EMVI evaluation; however, the evaluation of EMVI and MRF can be challenging in many cases (53).

The reduced territorial availability of MRI, higher costs, longer execution times, and limited patient characteristics (claustrophobia, marked obesity, metal devices implanted in the body, etc.) reduce the possibility of carrying out an MRI in all patients. On the other hand, the possible presence of marked colorectum stenosis makes the use of the EUS impossible. In these cases, computed tomography (CT) examination and subsequent SHV ​​evaluation become the first choice.

CT can be an alternative diagnostic imaging technique that allows to study of the entire abdomen and pelvis; CT is widely diffuse in clinical practice to assess the preoperative staging of abdominal lymphatic stations and distant metastases. CT is mandatory as 25% of patients affected by CRC have synchronous liver metastases (7, 54–56). Concerning the limited visualization of the mesorectal and the rectal wall, CT cannot be considered the gold standard, as it lacks of contrast resolution, especially for early-stage lesions confined to the rectal wall (32, 57, 58).

On the other hand, CT allows a clear visualization of the vascular anatomy. Concerning venous vascular system of rectum, the superior rectal venous plexus drains into superior hemorrhoidal vein (SHV), which has its origin in the hemorrhoidal plexus and, through this plexus, communicates with the middle and inferior hemorrhoidal veins. The superior rectal vein leaves the lesser pelvis and crosses the left common iliac vessels with the superior rectal artery and is continued upward as the inferior mesenteric vein and finally in the portal vein.

Many diseases are associated with focal or diffuse vascular enlargement of pelvic vessels, among which are pelvic tumors (59). In patients with CRC, it seems to be a variation in the splanchnic circulation. In particular, the SHV ectasia seems to be related to the extramural spreading of the tumor, being a new important negative prognostic factor (60).

This study aims to evaluate the correlation between the SHV ectasia, metastasis, and MRF invasion in the preoperative staging of rectal cancer.

## Materials and methods

### Image acquisition

Between January 2018 and April 2022, all consecutive patients with histopathological diagnosis of rectal cancer were enrolled at the Polyclinic of Bari, Italy, and their data were retrospectively analyzed.

Inclusion criteria:

- diagnosis of rectal cancer;

- informed signed consent to the use of their anonymous data for scientific research; and

- no sign of portal hypertension, cirrhosis, pelvic masses, and splanchnic vein thrombosis (59).

Exclusion criteria:

- any sign of portal hypertension, cirrhosis, pelvic masses, and splanchnic vein thrombosis; and

- lack of consent to participate to the study.

All patients underwent CT examination within 15 days before surgery and histopathological diagnosis as indicated by the standard of care of our institution.

All patients underwent multidisciplinary team discussion before treatment.

CT exams were obtained with a 320-row CT scanner (Multidetector CT Aquillon, Toshiba Medical System, Tokyo, Japan; detector collimation, 0.5 mm; increment, 0.5; 120/87 kVp/mAs).

CT protocol included a non-enhanced scan followed by multiphasic acquisition after the intravenous injection of 1.5 mL/kg of Iopromide (370 mgI/mL) at 2.5 mL/s through the ante-cubital vein using an automatic power injector. The patients were scanned in supine position.

The acquisition was performed from the diaphragm to the pubic symphysis in the non-enhanced and arterial phases; in the portal venous phase, the scan was extended to the thorax. No bowel preparation was performed before CT examination (61).

All CT data were transferred to a workstation equipped with dedicated software for image reconstructions (Vitrea FX 4.1, Vital Images, Minneapolis, Minnesota, USA).

### Dataset

Patients underwent surgery following the Italian National Guidelines (Italian Association of Medical Oncology (AIOM)) (62) with curative intent within 3 weeks from CT examination; then, the surgical specimens were submitted to the pathology department for examination. For each patient, we analyzed cancer location (low, middle, and high) and TNM parameters according to the VIII edition of the TNM classification by AJCC (22).

The present retrospective clinical study complied with ethical principles, including the Declaration of Helsinki of the World Medical Association and the additional requirements of Italian law and our Institutional Ethical Committee. In addition, the study was considered free from ethical review as it carries only negligible risk and involves the use of existing data, which contains only non-identifiable human data. The patient signed a written informed consent form approved by the local ethical board.

Preoperative CT scans were examined by two blinded radiologists with 10-year experience in gastrointestinal and oncologic radiology.

According to the literature, the cutoff value for SHV diameter used is 3.7 mm (60); the diameter was measured at the level of S2 vertebral level during portal venous phase after *4× image zoom* to reduce the interobserver variability (61). SHV was detected in all patients.

The parameters evaluated were as follows:

- tumor size and location: location of rectal cancer is classified in a cranio-caudal direction basing on the distance of the tumor from the anal verge as low (up to 5 cm), middle (from >5 cm to 10 cm), or high (from >10 cm up to 15 cm);

- detection of MRF infiltration, defined as the distance < 1 mm between the tumor margins and the fascia (26, 48, 63);

- SHV diameter;

- detection of mesorectal perilesional lymph nodes; and

- detection of metastasis.

### Statistical analysis

The baseline characteristics of the patients, the SHV ectasia, the presence of synchronous metastasis at CT examination, and the presence of lymph nodes involvement at pathological examination were evaluated by descriptive statistics. The relationship between SHV ectasia and the disease progression was evaluated through the Chi-square test or the Fisher test. P-value was judged statistically significant when less than 0.05.

The interobserver agreement was evaluated by using Cohen’s kappa (K). k > 0.81 assessed an almost complete agreement, and 0.61 < k < 0.8 and 0.41 < k < 0.6 assessed a substantial and a moderate agreement, respectively.

The statistical analysis was performed by using NCSS2007® software.

## Results

Forty-six patients were included in our study: 20 men (43.48%) and 26 (56.52%) women with a median age of 62 years. Descriptive statistics of pre-operative staging show that 16/46 (34.78%) patients had low rectal cancer, 18/46 (39.13%) patients had medium rectal cancer, and 12/46 (26.09%) patients had high rectal cancer.

Twelve of the 46 (26.09%) patients had synchronous metastatic involvement at the time of diagnosis of primary tumor.

Neoplastic infiltration of MRF was found in 4/46 (8.69%) patients: None of these patients presented hepatic metastasis. No lung metastases were detected in any patient.

Thirty-one of the 46 (67.39%) patients were SHV positive.

All patients undergoing surgery did not show any MRF infiltration.

At CT examination, 30/46 (65.21%) patients had a suspicion of perirectal lymph nodes.

Postoperative staging of patients undergoing surgery with neoadjuvant radiotherapy with or without chemotherapy after a minimum of 18 months of follow-up shows that 8 of the 31 patients who were SHV positive and M0 developed liver metastasis.

### SHV ectasia

**Figure 1:**
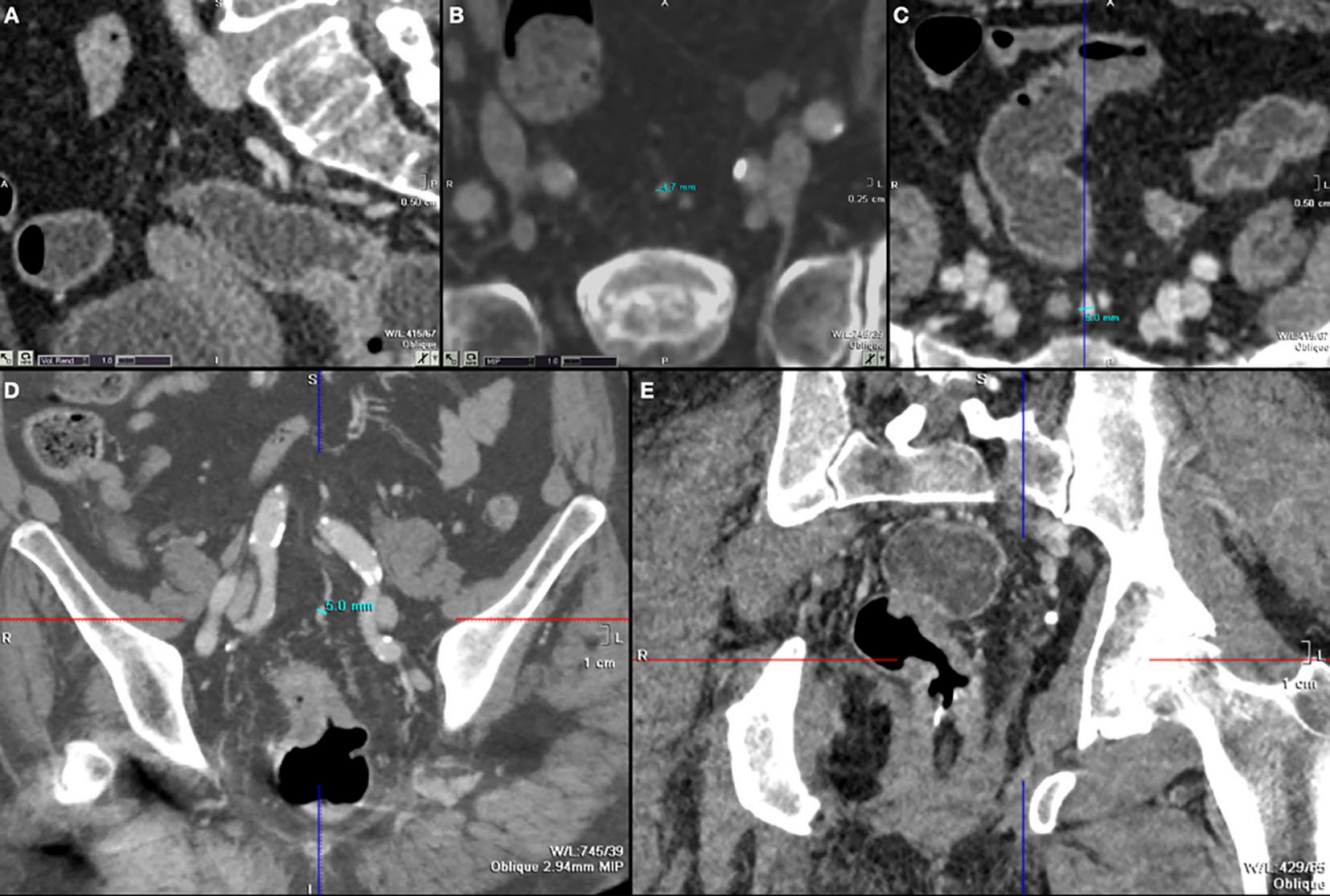
**(A)** S2 plane to evaluate SHV and S2 (sagittal reconstruction); **(B–D)** cases of SHV ectasia seen axial plane **(B, C)** and coronal plane **(D)**; **(E)** tumor of left lateral wall with MRF invasion and SHV ectasia.

The radiological evidence of SHV ectasia was shown in **Figures 1A–E**. Cohen’s kappa (K) was 0.78, indicating a high grade interrater agreement among the two expert radiologists.

All patients with MRF infiltration (4/46, 7.14%) presented SHV ectasia (average diameter of 4.4 mm).

**Table 1** shows that 67.39% (31/46) of patients had SHV ectasia. SHV ectasia was significantly related with the development of liver metastases at the moment of primary staging and during follow-up.

**Table 1:** Relationship between N and M status and SHV diameter.

	SHV−	SHV+	P-value
M−	12	14	0.031
M+	3	17	

## Discussion

In our experience, we evaluated the relationship between SHV diameter and T parameter, lymph node involvement, distant metastasis, and MRF infiltration. SHV ectasia may be related to metastasis development.

We found a significant relationship between SHV and advanced disease and disease progression. Hence, in further studies considering our preliminary data, SHV should be considered in the preoperative staging to better stratify the risk classification of disease progression. Our suggestion is to detect the high-risk patient group to perform a more intensive follow-up integrated with liver MRI that can more accurately detect and characterize also small potential liver lesions.

However, it should be underlined that venous vessel enlargement could be due to three principal mechanisms: increasing of venous drainage associated to neoplastic hypervascularization (64), splanchnic vein arterialization due to arterio-venous shunt, and increasing of venous pressure in neoplastic thrombosis (65). Considering this possible bias in patient selection, we preliminarily excluded from this study patients with cirrhosis, portal hypertension, pelvic masses, and splanchnic vein thrombosis, because SHV ectasia is frequent in these patients due to the presence of collateral circulation (59). Patient’s prognosis was affected by tumor invasion of the rectal wall, N stage, and MRF involvement (66, 67).

Following other literature experiences, we chose the cutoff of 3.7 mm to determine SHV ectasia. Some authors established that those patients with SHV diameter equal to or more than 3.7 mm had LVI (26, 60).

The nodal stage is often a challenge for radiologists especially because preoperative staging CT has a limited value in predicting lymph node metastasis in early rectal cancer and it is strongly related to metastatic disease and the treatment (6, 14, 25, 68–74). The study population showed that SHV diameter exceeded the cutoff by 3.7 mm in 79% of patients who had N+ confirmed after surgery and pathological examination. About distant metastasis, 75% of patients with liver metastasis had a SHV enlargement. Thus, patients with SHV diameter equal to or more than 3.7 mm tended to have nodal and distant metastasis.

In addition, we observed that, in the 16 patients who underwent neoadjuvant therapy, 3 did not show SHV ectasia although they had advanced cancer disease. Out of these three patients, two had low rectal cancer, and one had middle rectal cancer. We supposed that a lower rectal cancer, next to the anal verge, could have a different cancer venous vascular drainage, as inferior and/or middle hemorrhoidal vein that could justify no enlargement of SHV.

MRF is considered involved when the distance between the tumor and MRF is ≤1 mm. In our study, all patients with MRF involvement had SHV ectasia; this suggests a possible correlation between these two factors, both predictive of major invasion of the tumor.

Our experience confirms that SHV ​​diameter measurement could be a meaningful tool to analyze LVI, as previously demonstrated in other reports (26, 75). Furthermore, the study suggests that SHV ​​diameter could be a potential marker of MRF involvement.

If this were to be confirmed by further studies, then SHV ectasia may be integrated into the standardized parameters of the structured reporting (SR) for rectal cancer staging. The implementation of SR is important to offer referring physicians and patients an optimal quality of service and to provide researchers with data of the best quality (76, 77).

Obviously, we have to underline that MRF involvement and SHV diameter are useful only if integrated to the standard procedures concerning diagnosis and treatment of rectal cancer.

Nowadays, MRI is the most accurate non-invasive imaging modality to assess local staging at the moment of primary diagnosis (78–80).

MRI, through fast spin echo T2-weighted (FSE T2W), diffusion weighted imaging (DWI), and Apparent diffusion coefficient (APC) sequences, allows to recognize locally advanced diseases suitable of neoadjuvant CRT and to identify poor prognostic factors (81–83).

The identification of a locally advanced disease is mandatory to select the most precise treatment strategy, as the 25% of patients develop local recurrence after surgery and to improve the quality of life after surgery (14, 24, 44, 58, 84, 85).

The differentiation between T2 and T3 needs the MRI and the endorectal US in selected patients (86).

However, MRI has a high risk of over-staging disease due to the modification of muscolaris propria related to penetrating vessels or tissue desmoplastic reaction into the mesenteric fat (49, 87–89).

MRI is also useful for studying the locoregional nodal involvement and the extra-mesorectal lateral nodes, which, if pathological, makes the patients suitable for neoadjuvant chemotherapy (74).

MRI sensitivity is approximately 85% in nodal characterization; however, malignant cells might also be present in nodes < 5 mm of short axis, so our diagnosis power is still lower than our desire (83, 90).

All cases are characterized by a locally advanced disease diagnosed at MRI scan, and, in patients with a middle-low rectal tumor, the neoadjuvant treatment is mandatory before surgery, allowing organ-sparing surgical procedures with lower recurrence rates (87, 91, 92).

Therefore, for the local staging, MRI is the most complete diagnostic modality as it allows to accurately evaluate tumor location, Circumferential resection margin (CRM) involvement, nodal involvement, tumor deposits, or EMVI (93, 94).

Obviously, after any local treatment, it is also considered the gold standard for the restaging to assess the response to therapy (95–97).

At the same time, contrast-enhanced CT of the whole body is mandatory to detect distant metastases and complete the M-staging even in the pre-operative time even after neoadjuvant therapy (98, 99).

The diagnostic performance of CT of liver metastases is high, but it decreases for the lesions < 10 mm (56). In these cases, a diagnostic integration with liver MRI should be performed in selected patients (100, 101).

For this reason, several studies are focusing on the identification of the high risk patients (102–104).

Surely, rectal MRI allows to identify some negative features, such as EMVI, which is related to a higher incidence of developing distant metastases, particularly liver metastases (105–107).

Thus, taking into account what discussed above, the presence of SHV ectasia could also be considered a prognostic feature, suggesting the need of an MRI follow-up (60).

However, this tool should be validated in clinical practice in randomized prospective studies. In addition to radiological imaging, several studies are proposing liquid biopsy to detect circulating DNA that can contribute to the risk stratification of patients affected by CRC (108, 109).

In the era of precision medicine, liquid biopsy associated to imaging features could ensure a personalized follow-up or treatment strategy for different patients (110, 111).

Furthermore, radiomics tools have been proposed to analyze both the primary tumor and the most common site of metastatization.

In particular, many studies focused their attention on liver metastases, not only predicting genetic mutations on liver lesions but also predicting the future development of metachronous liver metastases in apparently healthy liver parenchyma (112, 113).

Currently, both liquid biopsy and radiomics have not already been validated in clinical practice due to the lack of prospective studies on multicentric cohorts; therefore, the analysis of the radiological features can be useful to create the first hybrid tools to create more intensive follow-up for high-risk patients. A more intensive follow-up can identify earlier patients affected by liver metastases and treat them with chemotherapy regimens.

This study has some limitations such as the small number of patients and, overall, the impossibility of comparing CT results with MRI data.

## Conclusion

SHV ectasia may be related to metastasis and MRF involvement; a cutoff of 3.7 mm in diameter is considered significant in our experience according to the literature. Therefore, SHV diameter could become an interesting tool to complete the preoperative staging and follow-up of rectal cancer.

## Data availability statement

The datasets presented in this article are not readily available because the raw data supporting the conclusions of this article will be made available by the authors, with undue reservation. Requests to access the datasets should be directed to dottchiaramorelli@gmail.com.

## Ethics statement

The studies involving humans were approved by University of Bari Medical School “Aldo Moro”. The studies were conducted in accordance with the local legislation and institutional requirements. The participants provided their written informed consent to participate in this study. Written informed consent was obtained from the individual(s) for the publication of any potentially identifiable images or data included in this article.

## Author contributions

NL: Writing – original draft, Writing – review & editing. AM: Writing – original draft, Writing – review & editing. NM: Conceptualization, Data curation, Formal analysis, Funding acquisition, Investigation, Methodology, Project administration, Resources, Software, Supervision, Validation, Visualization, Writing – original draft. CM: Methodology, Project administration, Resources, Software, Supervision, Validation, Visualization, Writing – original draft, Conceptualization, Data curation, Formal analysis, Funding acquisition, Investigation. RC: Conceptualization, Data curation, Formal analysis, Funding acquisition, Investigation, Methodology, Project administration, Resources, Software, Supervision, Validation, Visualization, Writing – original draft. SB: Conceptualization, Data curation, Formal analysis, Funding acquisition, Investigation, Methodology, Project administration, Resources, Software, Supervision, Validation, Visualization, Writing – original draft. PA: Software, Visualization, Conceptualization, Data curation, Formal analysis, Funding acquisition, Investigation, Methodology, Project administration, Resources, Supervision, Validation, Writing – review & editing. DS: Conceptualization, Data curation, Formal analysis, Funding acquisition, Investigation, Methodology, Project administration, Resources, Software, Supervision, Validation, Visualization, Writing – original draft. SG: Conceptualization, Data curation, Formal analysis, Funding acquisition, Investigation, Methodology, Project administration, Resources, Software, Supervision, Validation, Visualization, Writing – original draft. AI: Conceptualization, Data curation, Formal analysis, Funding acquisition, Investigation, Methodology, Project administration, Resources, Software, Supervision, Validation, Visualization, Writing – review & editing.
